# Survival in Malignant Peripheral Nerve Sheath Tumours: A Comparison between Sporadic and Neurofibromatosis Type 1-Associated Tumours

**DOI:** 10.1155/2009/756395

**Published:** 2009-04-07

**Authors:** D. E. Porter, V. Prasad, L. Foster, G. F. Dall, R. Birch, R. J. Grimer

**Affiliations:** ^1^Department of Orthopaedic Surgery, University of Edinburgh, Royal Infirmary of Edinburgh, Little France Crescent, Edinburgh EH16 4SU, UK; ^2^The Royal Orthopaedic Hospital, Bristol Road South, Northfield, Birmingham B31 2AP, UK; ^3^Peripheral Nerve Injuries Unit, Royal National Orthopaedic Hospital Trust, Brockley Hill Stanmore, Middlesex HA7 4LP, UK

## Abstract

We studied 123 patients with malignant peripheral nerve sheath tumours (MPNSTs) between 1979 and 2002. However, 90 occurred sporadically whereas 33 were associated with neurofibromatosis type 1 (NF1). Survival was calculated using Kaplan-Meier survival curves and we used Cox's proportional hazards model to identify independent prognostic factors. A 5-year survival for 110 nonmetastatic patients was 54%; (33% NF1 and 63% sporadic *P* = .015). Tumour stage and site were significant prognostic indicators after univariate analysis. After multivariate analysis, however, only NF1 (*P* = .007) and tumour volume more than 200 m (*P* = .015) remained independent predictors of poor outcome. We recommend that NF1 be taken into account during MPNST staging. As the survival rate in the NF group was dependant on tumour volume, routine screening of these patients with FDG PET and/or MRI may be warranted, thereby staging and controlling them at the earliest possible opportunity.

## 1. Introduction

Malignant peripheral nerve sheath tumours (MPNSTs) are aggressive, locally invasive soft tissue sarcomas, typically presenting as a rapidly growing and painful lump. These tumours account for up to 10% of all soft tissue sarcomas [1] and are associated with poor prognosis unless wide excision of the tumour is undertaken before local invasion or distant metastasis can occur. The incidence of sporadic MPNSTs is low, with a lifetime risk of 0.001% [2, 3] but in association with the familial condition neurofibromatosis type 1 (NF1), where these tumours often arise from malignant transformation of a plexiform neurofibroma, the incidence is much higher. Evans et al. [4] estimate the lifetime risk of developing MPNSTs in the population of patients with NF1 to be as high as 13%. A number of studies have compared survival in sporadic and NF1-associated tumours [1, 4–9] but no consensus has been reached on whether NF1 is an independent poor prognostic factor or not.

Our study aimed to determine factors important to outcome in a large population of patients with MPNSTs from two United Kingdom centres for soft tissue tumour surgery.

## 2. Patients and Methods

The medical records from 135 patients diagnosed with MPNSTs treated between 1979 and 2002 at two UK centers were reviewed. In 12 patients, there was insufficient follow-up data and they were excluded; leaving 123 patients that had follow-up data from 6 months to 21 years and they were included in the analysis.

Patients with NF1 were identified by the presence of certain characteristic features based on the diagnostic criteria for NF1 [10] including features such as café au lait spots, Lisch nodules, multiple neurofibromata, and a positive family history. A statement in the patient's medical records of an NF1 diagnosis was accepted as sufficient evidence for that individual to be placed in the NF1 group.

Histopathologists sitting on the national musculoskeletal tumour panel confirmed the diagnoses of MPNSTs and used the Trojani system to histologicaly grade the tumours. The date of diagnosis taken to be the date of first biopsy or excision from which a histological diagnosis of MPNSTs was made.

Operation notes and histology reports were utilised to determine the extent of surgery and the margins achieved. For the purposes of this analysis, amputation or wide excision was deemed to give adequate clearance; marginal excision and debulking were deemed to give inadequate clearance margins. Chemotherapy and radiotherapy intents were documented and tumour size and volume were calculated using surgical or magnetic resonance imaging records.

Survival data was calculated using Kaplan-Meier curves and multivariate analysis was performed using Cox's proportional hazards model using the statistical package SPSS 13.0. The effect of each variable was compared to the effect of the group as a whole.

## 3. Results

**Figure 1:**
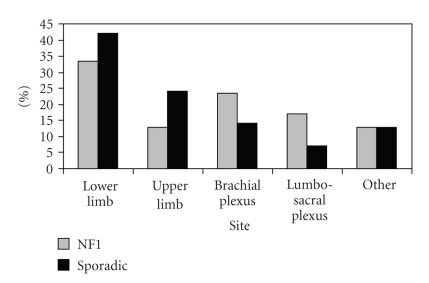
Tumour frequency by site.

Of the 123 patients in this study with MPNSTs, 33 patients (27%) had NF1. NF1 patients were significantly younger at diagnosis than those with sporadic tumours with a median age of 26 years compared with 53 years for sporadic MPNSTs, (*X* ^2^ = 23.65, *P* < .001). There were also significant differences in the distribution of the site of tumours between the two groups with relative overrepresentation of peripheral limb tumours in the sporadic group and axial tumours in the NF1 group (*X* ^2^ = 24.3, *P* < .001) (see Figure 1). There were no significant differences in the tumour volumes found in the NF1 and sporadic groups (*P* = .36).

**Figure 2:**
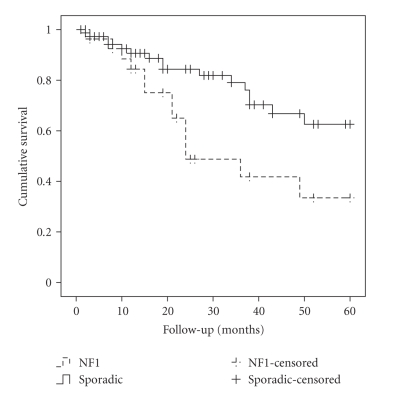
Kaplan-Meier survival in patients without metastases at diagnosis.

Overall 5-year survival for all 123 patients was 51% and was significantly worse for patients with NF1 than those with sporadic MPNSTs (32% versus 60%; *P* = .01). 13 patients (11%) had IUCC-TNM stage IV disease (metastases at diagnosis). Stage IV disease was more common in NF1 patients (15%) than those with sporadic tumours (9%) but NF1 was still associated with a significantly worse 5-year survival if patients with stage IV disease were removed from the analysis (33% versus 63%; *P* = .015) (see Figure 2).

The effect of other factors on survival in the group of patients without metastases at diagnosis was investigated using Kaplan-Meier analysis and is documented in Table 1.

Two factors remained significant on multivariate Cox regression analysis. Tumours with volume <200 ml had a significantly better prognosis (HR 0.355, 95% CI 0.15–0.82, *P* = .015) than larger tumours, and NF1 tumours were associated with significantly poorer prognosis compared with those occurring sporadically (HR 1.811, 95% CI 1.175–2.791, *P* = .007).

Local treatment included surgery in 94% and radiotherapy in 61%. Chemotherapy was given in 26%. 2/33 (94%) of the NF1 group and 5/90 (94%) of the non-NF1 group received surgery. 20/33 (65%) of the NF1 group and 55/90 (61%) of non-NF1 group received radiotherapy. The type of treatment had no significant effect on survival. Adequate excision margins were achieved in a similar proportion of NF1 and sporadic tumours (31% versus 28%). Local recurrence occurred in 24 patients. Where surgery was attempted, adequate surgical margins were achieved in 28% of patients, 6% of whom developed local recurrence. In the remaining 72% of patients in whom adequate surgical margins were not achieved, the local recurrence rate was 30%. This difference in local recurrence was statistically significant using the chi-square test (*P* < .001).

Although patients with local recurrence displayed a trend towards worse survival, this did not reach statistical significance. A trend was observed towards worse local recurrence-free survival in NF1 (5-year survival 70% versus 81% in sporadic tumours) but this did not reach statistical significance.

## 4. Discussion

Due to the relative rarity of MPNSTs, there have been few large studies into survival and those reporting 5-year survival lack consistency, with survival rates in the range of 39–85% [6, 11]. Our overall survival of 51% is within this range. There is a similar lack of consensus on the issue of whether or not NF1 is an independent indicator of poor prognosis. A number of studies report no significant difference between the 2 groups [1, 6, 11, 12]. Others, including this study report a poorer outcome in patients with NF1 [13–18]. It has been suggested that patients with NF1 are more likely to present late with MPNSTs because they may not be as concerned by the appearance of new swellings as the rest of the population. In our study, a greater percentage of NF1 patients had metastatic disease at presentation (15% versus 9%) but even with the exclusion of these cases from the analysis, our study of the remaining 110 patients demonstrated 5-year survival rates in NF1 patients only half as good as in patients with sporadic tumours. NF1 was also an independent predictor of poor prognosis on multivariate analysis. Possible explanations for the poorer prognosis seen in NF1 patients include differences in the genetic profile of tumours arising in these 2 groups [19–21] which might affect aggressive potential. Other cancers such as breast and ovarian cancers have also shown worse prognosis in familial cases compared to those occurring sporadically [20, 22].

Reports that patients with NF1 have an estimated lifetime risk of developing MPNSTs in excess of 10% [4], in conjunction with our findings of significantly poorer survival in NF1, underline the risk posed by these tumours, and the danger of complacency about new episodes of pain or swellings in these individuals.

This report clearly demonstrates that patients with NF1 are diagnosed with malignancy at a significantly younger age than in those with sporadic tumours and is consistent with other studies [3, 5, 9]. This reflects the nature of NF1 as a familial neoplastic trait that predisposes to both benign and malignant tumours. The NF1 gene was identified in 1987 [23] and functions as a tumour suppressor gene. Other familial neoplastic traits also exhibit age-dependent malignant change at a younger age than in the general population [20, 21].

On univariate analysis, the tumour volume, stage, and site were also found to be significant predictors of survival. Tumour volume was the only other factor that, together with NF1, remained significant on multivariate analysis. Histological grade was not found to correlate with survival; however, the result may have been skewed due to small numbers (15/129) of low-grade tumours. Recent published data from Hagel et al. [21] support our findings that the NF1 group is younger, has more axially located tumours, and has a worse prognosis. Interestingly, they presented evidence that the histopathology of NF1-associated tumours differs from the sporadic type. This may explain why we did not see a correlation between histological grade and survival. They postulated that and if a new grading system included NF1 as an independent prognosticator, then perhaps grade and survival would correlate.

There was no observable difference between tumour volumes in the sporadic and NF1 groups (*P* = .36). Independent of biology, a small volume tumour offers a better prognosis because of the higher chance of achieving wide resection margins.

We found that tumours affecting the peripheral portion of the upper limb were associated with the best survival on univariate analysis. Interestingly, tumours sited in the lumbosacral plexus also seemed to have a favourable prognosis. Since this group represents only 11% of the total, however, this finding should be interpreted with caution. Peripheral lower limb tumours accounted for the greatest proportion of tumours from the NF1 group (32%) and formed the majority (58%) of large volume tumours. These poor prognostic cofactors in our group of lower limb tumours cause the univariate site-specific survival differences to disappear on multivariate analysis. Other studies have reported that peripheral rather than centrally located tumours have better survival rates [11, 15]. This is likely to result from these tumours being more amenable to resection with wide margins or may be because they are detected earlier.

Recently, specialist centers have been using positron emission tomography to detect 18F-fluorodeoxyglucose (FDG PET) uptake in these tumours. Fisher et al. [24] showed that FDG PET is a useful tool in monitoring clinically stable NF1 patients with plexiform neurofibromas as it could predict which were more likely to subsequently grow rapidly. Also Brenner et al. [25] found that in NF1patients with MPNSTs, higher uptakes during FDG PET were associated with significantly worse survival whilst histopathological tumour grading did not predict outcome.

Definitive treatment for MPNSTs involves surgical removal of the tumour. Adjuvant or neoadjuvant therapy is increasingly considered but has not been shown to consistently improve survival [11, 26]. Only five patients in this study did not receive some form of surgical treatment.

It is well documented that these tumours can extend considerable distances along nerves and if suspected, a frozen section should be carried out at the proximal and distal limits of nerve resection to ensure clear margins. Adequate surgical margins were achieved in 31 out of 118 patients (26%) and only 6% of these patients developed local recurrence of their tumour, in contrast to 30% of patients in which clearance margins were deemed inadequate. When local recurrence did occur, this was associated with a worse outcome but the trend did not reach statistical significance. Other studies report that the failure to achieve local control of the tumour bears a major association with treatment failure and poor outcome [26, 27].

Any patient with an MPNST in association with NF1 should be carefully staged prior to treatment and should be managed by a multidisciplinary team familiar with both soft tissue sarcomas and NF1. In those patients who were treated with curative intent and had a marginal resection, recurrence rates remained low 3/32. Therefore, we recommend that postoperative surveillance should remain in accordance with current NICE sarcoma guidelines [28] and NF1 conference statement [10].

We conclude that as NF1 is an independent indicator of poor prognosis in MPNSTs, we recommend that this must be taken into account during the tumour staging. It may be necessary to have separate staging systems for sporadic and NF1-associated tumours to reflect this. As the survival rate in the NF group was dependant on tumour volume, routine screening of these patients with FDG PET and or MRI may be warranted, thereby staging and controlling them at the earliest possible opportunity.

## Figures and Tables

**Table 1:** Univariate analysis to determine factors significant for survival in patients without metastases at diagnosis.

Factor		NF1		Sporadic		All patients	
		5 year survival (%)	*P*	5 year survival (%)	*P*	5 year survival (%)	*P*
Stage	1	100		100		100	
	2	46.2	.375	76.1	.079	71.1	**.033**

Site	Lower limb	55.6		69.4		66.7	
	Upper limb	100		83.3		90.9	
	Brachial plexus	42.9		75.0		68.4	
	Sciatic plexus	50.0	.139	100	**.035**	88.9	**.036**

Volume	<200 ml	57.1		85.7		82.9	
	>200 ml	50.0	.119	66.7	**.015**	63.6	**.002**

Grade	Low	44.4		74.2		70.0	
	High	50.0	.862	79.2	.713	73.4	.606

Depth	Subcutaneous	50.0		90.0		83.3	
	Deep	50.0	.372	76.1	.755	72.2	.571
